# Enzymatic lignocellulose hydrolysis: Improved cellulase productivity by insoluble solids recycling

**DOI:** 10.1186/1754-6834-6-5

**Published:** 2013-01-21

**Authors:** Noah Weiss, Johan Börjesson, Lars Saaby Pedersen, Anne S Meyer

**Affiliations:** 1 Center for Bioprocess Engineering, Department of Chemical and Biochemical Engineering, Technical University of Denmark (DTU), Lyngby, DK-2800 Kgs, Denmark; 2 Novozymes A/S, Krogshøjvej 36, Bagsværd, DK-2880, Denmark

## Abstract

**Background:**

It is necessary to develop efficient methods to produce renewable fuels from lignocellulosic biomass. One of the main challenges to the industrialization of lignocellulose conversion processes is the large amount of cellulase enzymes used for the hydrolysis of cellulose. One method for decreasing the amount of enzyme used is to recycle the enzymes. In this study, the recycle of enzymes associated with the insoluble solid fraction after the enzymatic hydrolysis of cellulose was investigated for pretreated corn stover under a variety of recycling conditions.

**Results:**

It was found that a significant amount of cellulase activity could be recovered by recycling the insoluble biomass fraction, and the enzyme dosage could be decreased by 30% to achieve the same glucose yields under the most favorable conditions. Enzyme productivity (g glucose produced/g enzyme applied) increased between 30 and 50% by the recycling, depending on the reaction conditions. While increasing the amount of solids recycled increased process performance, the methods applicability was limited by its positive correlation with increasing total solids concentrations, reaction volumes, and lignin content of the insoluble residue. However, increasing amounts of lignin rich residue during the recycle did not negatively impact glucose yields.

**Conclusions:**

To take advantage of this effect, the amount of solids recycled should be maximized, based on a given processes ability to deal with higher solids concentrations and volumes. Recycling of enzymes by recycling the insoluble solids fraction was thus shown to be an effective method to decrease enzyme usage, and research should be continued for its industrial application.

## Background

Limited oil resources and the devastating effects of climate change from the burning of fossil fuels make it necessary to identify sustainable alternative sources of energy for the future [1]. Many alternative, potentially sustainable sources of energy exist, however there are limited choices for the replacement of liquid fossil fuels. One of these possibilities is the production of fuels from lignocellulosic biomass. The first step in one of the more promising conversion pathways includes a biochemical conversion of the lignocellulosic material whereby the structural sugars present in lignocellulosic biomass are depolymerized into their monomeric constituents via an enzymatic hydrolysis step, providing a fermentable sugar stream rich in glucose [2].

Recent commercial cellulase preparations have been shown to be effective at hydrolyzing cellulose under industrially relevant conditions, however the high cost of enzymes remains a significant barrier to the economical production of ethanol from lignocellulosic biomass [3,4]. It is therefore necessary to reduce the amount of enzyme required for the enzymatic hydrolysis step. Current enzyme loadings for cellulose hydrolysis remain high compared to enzymatic starch hydrolysis. Hence, reducing the amount of enzyme needed or increasing the enzyme productivity in the process is a promising approach. This has been investigated by looking for methods to improve hydrolysis yields while lowering enzyme doses. A variety of methods have been suggested to achieve increased hydrolysis yields, including via surfactant addition [5], gradual substrate loading [6] or advanced reactor configurations coupled with product removal to avoid inhibition [7-9]. However, these methods have yet to be shown to be cost effective. One method that may reduce the amount of enzyme used and increase enzymatic productivity is to recycle the enzymes [10,11]. Conceptually, the assumption is that by recovering the active enzymes from the output of the enzymatic hydrolysis step, it is possible to decrease the amount of new enzyme which must be added to the hydrolysis, and therefore reduce the overall enzyme cost in the process. This method also lends itself to continuous processes, a necessity for industrial application. Recycling can also increase the enzyme substrate interaction time, which can lead to an overall increase in enzyme conversion efficiency. Due to mass transfer limitations of the insoluble substrate, immobilization, the most commonly applied method of enzyme recycle, is not an option for cellulose hydrolysis, so other options for enzyme recycling must be developed.

The cellulase enzymes currently employed in the hydrolysis of lignocellulosic biomass readily bind to cellulose, and those enzymes which are active on the cellulose polymer, specifically cellobiohydro-lyases (EC 3.2.1.91) and endoglucanases (EC 3.2.1.4) remain adsorbed to the cellulose polymer during hydrolysis [12-14]. Because the substrate is present as a solid, the cellulases stay attached to the insoluble solids fraction. For cellulose biomass substrates that have been de-lignified during pretreatment, a significant amount of cellulase enzymes have been found to desorb from the solid substrate during the hydrolysis [13,15]. β-glucosidases (EC 3.2.1.21) have a soluble substrate, cellobiose, and a majority of the enzyme activity has been found to remain in the soluble (liquid) fraction during hydrolysis [13]. Cellulases also adsorb to lignin, and therefore a significant fraction can be found adsorbed to the lignin throughout the reaction, as lignin concentration remains constant [16]. Thus the enzymes are primarily associated with the insoluble solids fraction, though a significant amount of activity, especially β-glucosidase, can still be found in the liquid fraction. To successfully recover the enzymes, they must either be separated and collected from their associated fractions, or the enzyme containing fractions must be recycled into the subsequent hydrolyses.

A significant amount of work has already been carried out investigating the recycle of cellulases [10,11,14,17-21]. The majority of cellulase recycling methods that have been reported involved either separating the enzymes from the solid or liquid phases, or recycling of the solid and/or liquid phase directly. Approaches have been demonstrated where free enzymes were recovered from the liquid fraction by membrane filtration [11], or where fresh substrate was introduced to the liquor fraction and the enzymes were allowed to adsorb to the substrate before a further separation and hydrolysis step [18,19]. Similarly, the enzymes associated with the solids have been recovered by washing with excess volumes of buffer, sometimes with surfactants, to desorb the enzymes, which were then concentrated and added to the fresh substrate [19]. These methods have shown varying levels of success under controlled laboratory conditions and with specially prepared feedstocks. The recycle methods which rely on enzyme isolation and recovery have yet to be demonstrated under process relevant conditions, and the effective scale up of the separation processes used have not been shown. A more straightforward approach to enzyme recycle is the direct recycle of the residual solid fraction into subsequent hydrolyses. The recycle of the solids fraction after solid liquid separation has been demonstrated on a number of substrates and in combination with other recycle methods [15,19,22]. This approach has also been coupled with methods for recovering enzymes from the liquor fraction, primarily by the method of a limited exposure time of the liquor to fresh substrate at low temperatures [18,19]. Previous attempts have shown that a large portion of the enzymatic activity could be recovered using a combination of these methods. However, these studies relied on high enzyme loadings, which often resulted in complete hydrolysis of the cellulose, low total solids concentrations (from 2-5% TS), extended reaction times, and supplementation of each recycle round with β-glucosidase [14,15,18,19,22]. As well, the best results were demonstrated on pretreated materials with very low lignin content, which were produced using pretreatments specifically designed to remove lignin [18,19,22]. Recycle performance decreased significantly when applied to lignin containing substrates created with more standard pretreatment [18]. While these studies have shown that enzyme recycle is technically possible under defined conditions and with ideal enzyme extraction methods, no work has yet demonstrated cellulase enzyme recycle under process relevant conditions and by relying on unit operations which could be economically feasible for industrial production. Little consideration has been made into the process implications of enzyme recycle on an industrial scale, and how various methods of enzyme separation or re-adsorption could be applied. Most recycle studies have attempted to maximize the amount of activity recycled, irrespective of process intensity, with the idea that recovery must approach 100% of initial activity to be industrially interesting. Compared to the current cellulose hydrolysis processing regimes for lignocellulosic biomass, which requires fresh enzyme addition with each new batch of substrate, a significant fraction of enzyme activity could be recycled with minimal sample processing, and large decreases in enzyme cost could be achieved.

The objective of this study was to determine if, by recycling the insoluble solids fraction, a significant amount of enzyme activity could be reused, and therefore increase overall product yields or decrease the amount of required enzyme needed to reach a given level of conversion. It was also desired to determine which process variables, such as solids washing and fraction of solids recycled, had significant effects on process yields, and how the manipulation of these variables would impact the hydrolysis reaction conditions. In this study, the recycle of cellulase enzymes by recycling the insoluble solids residue present after hydrolysis was investigated and evaluated at conditions which were closer to industrial processing conditions than many previous studies. The efficacy of enzyme recycle by the proposed method was evaluated for a number of successive recycle scenarios while modifying a number of recycle conditions and measuring the changes in product formation. The data from these experiments were then used to develop a computational model to predict process parameters after a large number of recycles, thus giving an idea of steady state conditions. The influence of the lignin rich residue was also directly evaluated for its effect on enzyme hydrolysis.

## Results and discussion

### Ability of recycled cellulases to hydrolyze freshly added cellulose

**Figure 1:**
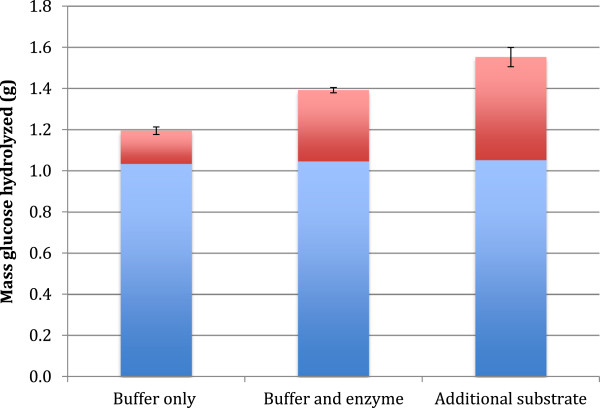
**Mass glucose produced for three different treatments of insoluble hydrolysis residue at an initial substrate concentration of 15% TS. **Bottom bars (blue) are mass of glucose produced in the first 72 hours, and top bars (red) are the mass produced after solid liquid separation and re-suspension of the insoluble solids with either buffer alone, buffer and enzyme, or buffer with 3 g of PCS substrate. Values are reported as the average of triplicate experiments. Error bars represent ± one standard deviation.

From examining the amount of glucose produced in the course of the reaction, it could be seen that significant amounts of glucose were produced from the fresh substrate when the insoluble solids were recycled (Figure 1). Glucose was produced in the second hydrolysis round under all three conditions, resuspension of the residual solids in buffer, resuspension of the solids in buffer with additional enzyme, and addition of fresh substrate to the residual solids, during the second 72 hour period (Figure 1). The amount of glucose produced when fresh substrate was added to the residual solids (0.50 g) was significantly larger than when either buffer alone (0.16 g) or buffer and additional enzyme (0.34 g) were added (Figure 1). This increased glucose production above what was produced from simply resuspending the remaining solids in new buffer or with fresh enzyme suggests that the enzymes which were associated with the insoluble fraction were capable of hydrolyzing cellulose from the fresh substrate.

### Process variables effect on recycle performance

**Figure 2:**
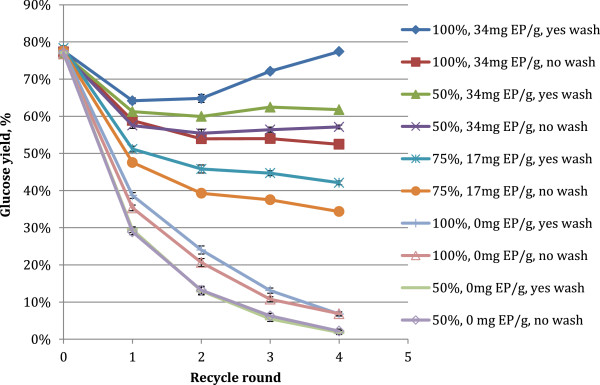
**Glucose yields from factorial recycle experiment. **72 hr reaction time between recycles, 10% TS substrate concentration, 51.4 mg EP/g cellulose initial loading. Conditions varied by fraction of insoluble solids recycled, solids washing between recycles, and the amount of additional enzyme added with each recycle. Reported values are averages of duplicates, error bars represent ± one standard deviation of the experimental center point.

In the first factorial experiment, the 72 hour glucose hydrolysis yield (recycle round 0) was 77% (w/w adjusted for the hydration factor, see Glucose yield section). Glucose yields after the first recycle round (additional 72 h) for each individual hydrolysis condition ranged between 30 and 65%, and between 2 and 77% after the fourth recycle round (Figure 2). Glucose yields decreased below the first hydrolysis round glucose yield (77%) for all conditions with each recycle. In only the highest response level condition (100% solids recycle, 34 mg enzyme product (EP)/g, washing of solids), did the glucose yield reverse a downward trend to increase to 77% for the fourth recycle round. It is speculated that this may be due to a buildup of active enzyme in the recycled fraction. Samples with no extra enzyme addition followed an exponential decay of glucose yields with subsequent recycle rounds, and those where makeup enzyme was added decreased at a slower rate or stabilized after the second recycle round. Statistical analysis showed that increasing all three experimental factors had significant positive effects on glucose yields, with the largest effect from increasing enzyme application, followed by the fraction of insoluble solids recycled, and finally the washing of the solids, which only had a slightly positive effect. The multivariate statistical analysis gives an overall effect across all the paired conditions including with and without the washing step. This overall effect may thus override pairs for which there was no effect of the washing, e.g. as in this case in the 50% solids recycle with no extra enzyme addition (50%, 0 mg EP/g, Figure 2).

The model was found to be statistically significant (P<0.0001) for the prediction of all responses, and actual versus predicted had r-squared values between 0.97 and 0.99. The increase in glucose yield for recycle round 2–4 obtained with recycling of 100% of the solid, washed residues, and enzyme supplementation (34 mg EP/g cellulose) might be a result of both increased enzyme levels due to high supplementation and recycle levels and the impact of the removal of glucose (product inhibitor) and putative inhibitors resulting from the pretreatment during the washing step. The results regarding washing of the substrate agree with data published by others. Washing has thus long been known to remove acid and any eventual inhibitory substances that inhibit cellulolytic enzymes from *Trichoderma sp*. [23]. More recently, Xue et al. [24] showed that a washing stage (in their case with buffer) together with addition of a surfactant during solids recycling, improved the hydrolysis efficiency with enzyme recycling on pretreated softwood and hardwood undergoing enzymatic hydrolysis [24].

**Figure 3:**
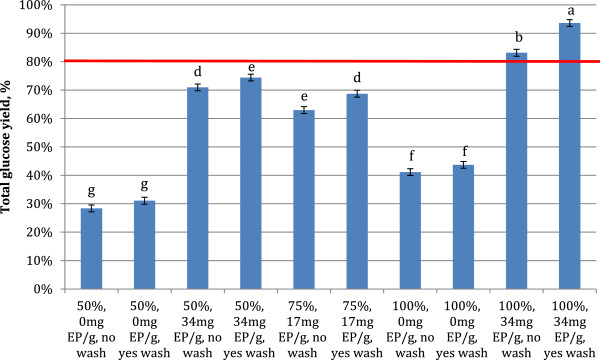
**Total glucose yields over the course of 4 solids recycles for factorial experiment. **72 hr reaction time between recycles, initial substrate loading of 10% TS, and initial enzyme loading of 51.4 mg EP/g cellulose. Averages of duplicates with standard deviation of 4 center points. Error bars represent ± one standard deviation. The horizontal red line represents the glucose yield at 51.4 mg EP/g cellulose at 72 hours with no recycle, the ‘break even point’. Roman letters a-g indicate significantly different values by ANOVA (95% confidence intervals, pooled standard deviation 0.799 (N=22)).

Total glucose yields ranged between 28 and 93% of total cellulose added during the experiment (Figure 3). Only two conditions had total glucose yields above the initial 72-hour hydrolysis condition, and these were the conditions with the highest level of makeup enzyme addition and insoluble solids recycle. For these conditions the makeup enzyme loading was 34 mg EP/g cellulose, and the fraction of solids recycled was 100%. Thus significantly higher total glucose yields were obtained when 33% less enzyme was applied in the recycle rounds than in the initial 72 h hydrolysis (34 mg is 67% of 51 mg applied initially), representing a significant improvement in process performance. When analyzed by least squares fit, the amount of makeup enzyme added and the fraction of insoluble solids recycle had significant (P<0.05) positive effects on the total glucose yield, with the former having the largest impact on total yields. No significant two factor interactions were identified.

Similar trends were observed for the total mass of glucose produced in each recycle round. Because of different levels of insoluble solids recycle, there were different amounts of cellulose present at the beginning of each hydrolysis. Experimental conditions which had makeup enzyme added showed a relatively constant amount of glucose produced in each recycle round past the first round (between 0.53 and 0.71 g), and under conditions with the highest makeup enzyme loading and solids recycle glucose production increased with each subsequent recycle, from 0.74 g to 1.02 g. This observation suggests that a constant amount of glucose production from recycle to recycle were obtained under recycle conditions with makeup enzyme loadings of 34 mg EP/g cellulose, even if the calculated glucose yields decreased for each individual recycle round (Figure 2).

**Figure 4:**
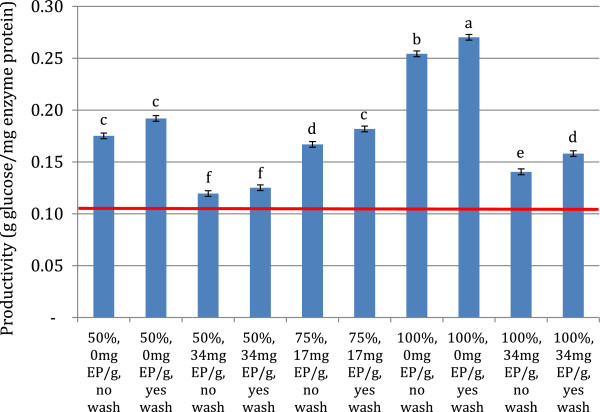
**Enzyme productivities of factorial recycle experiment samples. **Based on total glucose produced and the total mass of enzyme protein added over the course of the experiment. Averages of duplicate experiments and error bars represent ± one standard deviation of the center point. The red line represents the productivity for the 72 hour hydrolysis with a 51.4 mg EP/g cellulose loading. Roman letters a-f indicate significantly different values by ANOVA (95% confidence intervals, pooled standard deviation 0.0045 (N=22)).

Enzyme productivities ranged from between 0.12 to 0.27 g glucose/mg enzyme protein applied (Figure 4). In comparison, the 72 hour hydrolysis yield with an enzyme loading of 51.4 mg EP/g glucose had an enzymatic productivity of 0.097 g glucose/mg enzyme protein, and a 95% glucose yield with a 51.4 mg EP/g cellulose enzyme loading would have a productivity of 0.11 g glucose/mg enzyme protein. All recycle conditions increased the total enzymatic productivity within the system, and in the samples with the highest glucose yields, the productivity increased 50% above the non recycle scenario. Productivity decreased as the amount of makeup enzyme applied increased, but increased with increasing insoluble solids recycle. This was most likely due to the larger amounts of active enzyme and cellulose present with higher fractions of insoluble solid recycle, and therefore there were more cellulose and enzymes available for a longer time. Productivity did not correlate with glucose yields, and therefore on its own was not a good indicator of best process performance, however the recycle of enzyme showed significant increases in productivity and enzyme efficiency for all recycle conditions.

The most significant factors affecting the performance of enzyme recycle was the amount of additional enzyme applied, and the fraction of solids recycled. The washing of the solids was determined to not have an overall significantly positive effect on recycle performance. Initial glucose concentrations varied depending on how much of the non-washed residue was recycled. At the beginning of the recycle experiment, initial glucose concentrations were ~5 g/L from the pretreated slurry, and in some cases reached 20 g/L in the beginning of the 4^th^ recycle round for conditions with 100% recycle (data not shown). Washing resulted in lower glucose concentrations in the hydrolysate, which is not desirable for industrial scenarios. Coupling the non-significant impact of washing on total glucose yields with expenses that would be incurred by including a washing step in an industrial process, it was decided that solids washing was not beneficial to the recycle process. Therefore, the investigation of washing as an alternative for improving recycle performance was not continued.

### Recycle at higher solids concentrations with response surface methodologies

**Figure 5:**
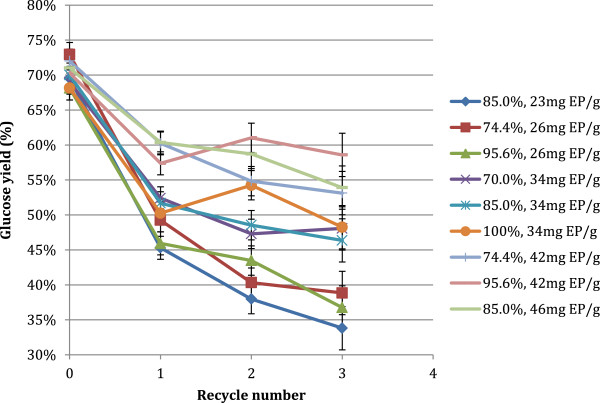
**Glucose yields as fraction of total theoretical glucose production after each recycle round on PCS. **72 hr reaction time between recycles, initial 15% TS loading, and 51.4 mg EP/g cellulose initial enzyme loading. Reaction conditions varied by the fraction of insoluble solids recycled (%), and the amount of makeup enzyme added per gram cellulose added with each recycle (mg EP/g). Values are averages of duplicate experiments. Error bars are ± one standard deviation of 6 center point experiments.

When the initial % TS concentration (w/w) was increased from 10% to 15%, the glucose yield achieved after the initial 72 hours decreased from 77% to 69% w/w (Figure 5). All of the experimental conditions showed a decrease in theoretical glucose yields after the first recycle, but many conditions produced relatively constant yields after the first initial drop in yields (Figure 5).

It is well documented that enzymatic cellulose hydrolysis of lignocellulosic substrates produces a negative relation between glucose yields and substrate concentration, whereas there is a positive relationship between the final glucose concentration and the substrate load [6]. This paradox has been ascribed to be due to non-productive enzyme adsorption to the solid substrate [2,16,21], but may also be a result of product inhibition by glucose on the cellulases, in particular when the glucose product levels become higher than 10 g/L [8]. The highest yields after the third recycle were obtained in the conditions with the highest enzyme dosage and fraction of solids recycled. The fractional decrease in yields for each recycle were similar to the 10% TS experiments for the same makeup enzyme loading and fraction of solids recycled. The least squares fit model applied to the data was found to be statistically significant (P<0.0001) for the prediction of all responses, and actual versus predicted data had r-squared values between 0.94 and 0.98. Both factors were again found to have a significant effect on all measured responses.

**Figure 6:**
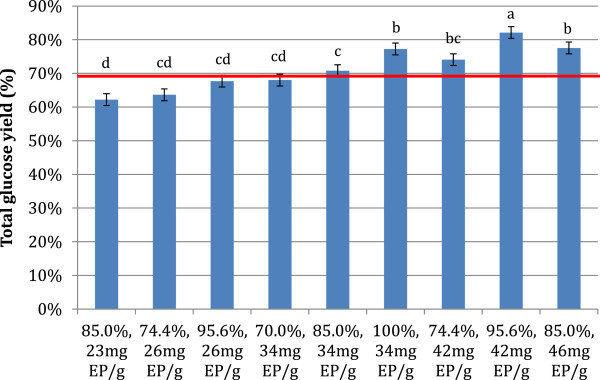
**Total glucose yields, as fraction of theoretical maximum glucose produced, for 4 rounds of hydrolysis for the response surface experiment on PCS substrate. **72 hr reaction time between recycles, initial 15% TS loading, and 51.4 mg EP/g cellulose initial enzyme loading. Reaction conditions varied by the fraction of insoluble solids recycled (%), and the amount of makeup enzyme product added per gram cellulose added with each recycle (mg EP/g). Values are averages of duplicate experiments. Error bars are ± one standard deviation of 6 center point experiments. The red bar represents the glucose yield at 72 hours with no recycle and 51.4 mg EP/g cellulose. Roman letters a-d indicate significantly different values by ANOVA (95% confidence intervals, pooled standard deviation 1.722 (N=22)).

Total glucose yields after 3 recycle rounds ranged from 62% to 82% (Figure 6). A number of conditions performed better than the 72 h hydrolysis yields, with the highest yield from the condition with 95.6% insoluble solids recycle and makeup enzyme of 42 mg EP/g cellulose. The center point of the experiment matched the yields of the 72 h hydrolysis. Thus by recycling only 85% of the insoluble solids it was possible to decrease the enzyme loading by 33% and maintain the same level of cellulose hydrolysis. The amount of glucose produced during each recycle round stayed relatively constant for most experimental conditions, or increased from 1.12 g to 1.45 g for the most favorable conditions (data not shown), and relative trends were similar to those for glucose yields.

**Figure 7:**
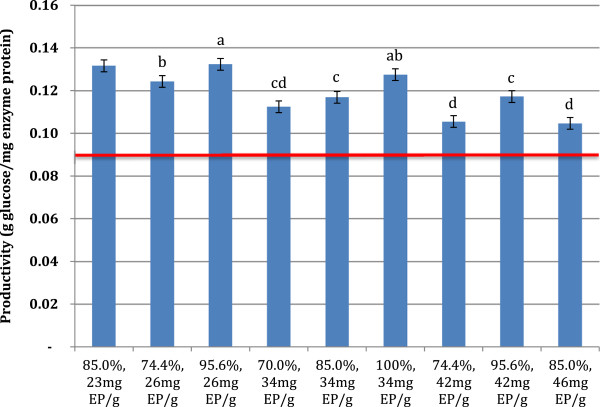
**Enzyme productivity over the response surface experiment on PCS2. **72 hr reaction time between recycles, initial 15% TS substrate loading, and 51.4 mg EP/g cellulose initial enzyme loading. Reaction conditions varied by the fraction of insoluble solids recycled (%), and the amount of makeup enzyme added per gram cellulose added with each recycle (mg EP/g). Values are averages of duplicate experiments. Error bars are ± one standard deviation of 6 center point experiments. The red line represents the productivity of the 72 hour hydrolysis yield with no recycle and 51.4 mg EP/g cellulose. Roman letters a-d indicate significantly different values by ANOVA (95% confidence intervals, pooled standard deviation 0.0027 (N=22)).

The productivity for the 72 h hydrolysis was 0.086 g glucose/mg enzyme protein applied. All recycle conditions had higher productivities than the 72 h hydrolysis conditions, and these productivities primarily were inversely proportional to the amount of makeup enzyme used (Figure 7). Productivities ranged from 0.11 to 0.13 g glucose/mg enzyme protein. This again showed a significant increase in enzyme productivity and utilization for cellulose hydrolysis. Thus, trends which were present at 10% TS were also observed at higher solids concentrations. These results point to maximizing both makeup enzyme loading and the amount of solids recycled to increase recycling efficiency and to boost glucose yields. However, it is not possible to recycle 100% of the insoluble stream on an industrial scale, as this would lead to an infinitely large reactor and reaction prohibitive solids concentrations, and increasing enzyme loading significantly would lead to increases in operating costs. Thus these results must be incorporated with other processing parameters to determine optimal operating conditions for an industrial recycle scenario.

### Modeling of recycle reaction conditions

**Figure 8:**
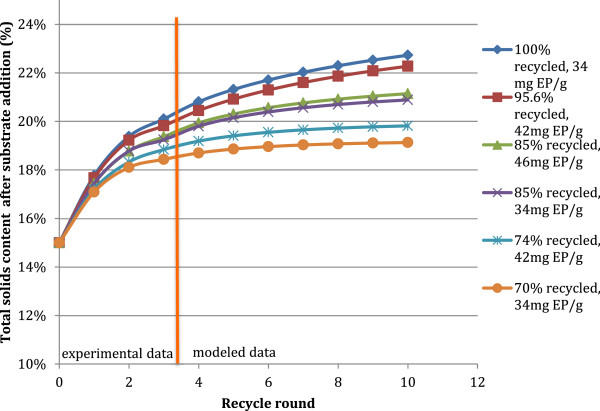
**Total solids content (%TS) of hydrolysis reactions with progressive recycles for different solids recycle and enzyme loading scenarios. **Experimental data used in the model is from response surface experiment on PCS2 with an initial solids loading of 15% TS, 3 total recycles. Data shown is for selected experimental conditions.

Based on the performance and gravimetric data from the recycle experiments, it was possible to construct a mathematical model to determine process parameters for an extended number of successive recycle rounds. Of specific interest was the effect of recycle on the TS content of the total reaction mass (Figure 8), and the composition of the insoluble solids. The TS content increased in all of the recycle scenarios significantly with subsequent recycles, and the TS content at the 10^th^ recycle ranged from 19 to 23% TS (data not shown). Solids concentrations can be a limiting factor in lignocellulosic processes, primarily due to difficulties in mixing, and it is therefore important to consider how any new recycle method would affect this variable [3]. Solids recycle had a significant impact on the TS content, and the negative impacts of increasing that solids concentration must be weighed against any improvement in enzyme performance due to the recycle. However, the rheological properties of lignocellulosic biomass has been found to change depending on the degree to which the cellulose has been hydrolyzed [6] and thus the mix of pretreated biomass with recycle residue could be expected to exhibit different mixing characteristics than pretreated biomass alone.

**Figure 9:**
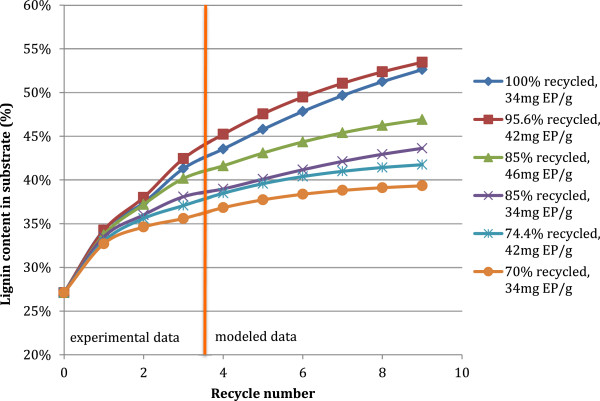
**Predicted lignin content of insoluble solids for successive recycles of insoluble solids**, **for different fractions of solids recycle and makeup enzyme addition. **Experimental data used in the model is from response surface experiment on PCS2 with an initial solids loading of 15% TS, 3 total recycles. Data shown is for selected experimental conditions.

The lignin content of the insoluble substrate was shown to increase significantly with subsequent recycles in the model, increasing from a starting lignin content of 27% to between 39 and 53% of total insoluble solids (Figure 9). The increase in lignin content depended primarily on the fraction of solids recycled and on the enzyme loading. Due to that more cellulose was hydrolyzed with increased enzyme loadings, the lignin content in the insoluble solids fraction which remained after each hydrolysis increased proportionally and thus the residual insoluble material had higher lignin content when recycled. The lignin was in this model regarded as a nonhydrolyzing material and was therefore assumed to stay in the solid fraction.

Similar trends to those exhibited by total solids concentrations in Figure 8 were seen for the total mass of the reaction, which was found to increase as a function of the fraction of solids recycled. From an initial mass of 20 g, the total mass of the reaction with a 70% solids recycle increased to 33 g by the 10^th^ recycle (data not shown). This significant increase in reaction mass, and with it reaction volume, implies that any recycle method would require significantly larger equipment for the hydrolysis, and thus increased capital and operating costs. The starting cellulose composition of the insoluble substrate was found to decrease with subsequent recycles, and trended inversely to the lignin content of the insoluble substrate. The cellulose content of the insoluble fraction decreased from 63% to between 44 and 23% by the 10^th^ modeled recycle round, depending on the fraction of insoluble solids recycled and the enzyme dosage.

### Effect of increased concentrations of lignin residue on cellulose hydrolysis

**Figure 10:**
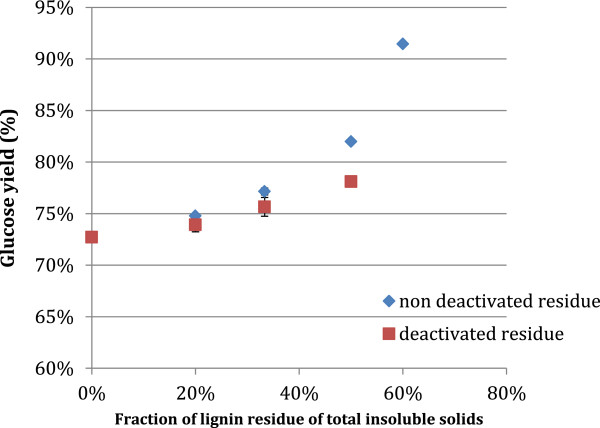
**Theoretical glucose yields for 72 hr hydrolysis reactions on assay PCS material. **Initial total solids concentration of 5-7% w/w. Enzyme loading of 34 mg EP/g cellulose in assay substrate. Lignin residue added was approximately 95% lignin and ash, and 5% cellulose. “Fraction of lignin residue of total insoluble solids” refers to the percentage of the initial total insoluble solids, which was the lignin residue (total insoluble solids were added lignin residue and unhydrolyzed PCS). Residue was deactivated by heating to 100°C for 10 minutes in a block heater. Values are average of duplicate experiments. Error bars represent ± one standard deviation.

Based on trends suggested by the model that show an increase in lignin in the insoluble fraction, it was desirable to determine the direct effect of this phenomenon on hydrolysis performance. It was found that for low solids hydrolysis conditions, increasing the fraction of lignin in the insoluble solids fraction did not have a negative effect on glucose yields (Figure 10). Glucose yields were found to increase slightly both in the presence of non-deactivated and the deactivated lignin rich hydrolysis residue, although to a lesser extent for the latter. These data differ significantly from what would have been expected, as lignin is known to nonspecifically bind cellulase enzymes, and therefore to act inhibitory on cellulose hydrolysis [13,16]. This aspect has also been considered to be an issue in relation to enzyme recycling via recycling of lignocellulose solids [10]. The observed result may be related to the specific nature of the lignin rich hydrolysis residue used to increase the lignin content of the reaction. The lignin residue was previously exposed to cellulase enzymes, and therefore the binding sites on the lignin, may already have been occupied by enzymes from the previous hydrolysis, and the substrate moreover had a particularly large surface area since it had been been milled (see Feedstocks and chemicals section). The method which was used to produce the lignin rich residue was identical to the standard enzymatic hydrolysis used in the study, and therefore it can be assumed that the recycled insoluble solids residue behaves similarly when it is recycled. It has already been shown that active enzymes remain associated with the insoluble fraction in this study, and therefore it is also feasible that inactivated enzymes might remain associated with the solids and occupy lignin-protein binding sites. The lack of decrease in glucose yields with the introduction of the enzyme deactivated lignin rich residues may analogously be due to the non-specific enzyme binding sites on the lignin residue being occupied by deactivated enzyme protein residues. The slight increase observed for the higher fractions of lignin residue (Figure 10), could be due to less glucose product inhibition of the enzymes due to the washing as well as a result of hydrolysis of the 5% residual cellulose accompanying the material (see Lignin residue hydrolysis response section). Nevertheless, these results show that the increase in the lignin content of the insoluble fraction during recycle does not have a negative effect on glucose yields.

### Evaluation of solids recycle for enzyme recovery and yield improvement

The recycle experiments demonstrated that a significant portion of the enzyme activity could be recycled with the insoluble solids fraction after each hydrolysis. Total enzyme productivity was increased for all of the recycle scenarios, showing that the enzymes were better utilized during the reaction. The insoluble solid recycle method also increased enzyme substrate contact time, as unreacted substrate was returned to the reaction. However, the amount of enzyme activity which was successfully recycled was significantly less than the total amount of enzyme which was initially added to the hydrolysis. This significant loss in overall enzyme activity may be due either to thermal deactivation and precipitation of the enzyme, increased nonspecific binding by fresh lignin, and/or loss of soluble enzyme in the liquor fraction. Specifically, a loss in β-glucosidase activity in the soluble fraction, which has been shown to be present in significant quantities during hydrolysis in the liquid phase [13], could upset specific cellulase activity ratios and lead to less than optimal performance of the enzyme preparation. Potentially, enzyme products or makeup enzyme products for biomass hydrolysis processes that includes recycling of solid substrate could be designed to match the specific enzyme activities that are being lost during recycling scheme.

The recycle of the insoluble solids caused large impacts on the physical and compositional parameters of the hydrolysis. Specifically, the recycle led to significantly increased total solids concentrations, total reaction masses, and to an increase of lignin in the solids composition. These increases limit the amount to which solids recycle can be applied in an industrial process, as a significant solids purge would need to be incorporated to achieve steady state and to maintain acceptable operating conditions. As the ability to recycle enzyme activity was related directly to the amount of solids which could be recycled, the ability to recycle the enzymes would be effectively limited by the need for this solids purge. Determining the optimal tradeoff between recycling enzyme activity and increased operating solids concentration is highly process dependent, and is beyond the scope of this investigation. The increase in solids concentration due to recycling could also lead to decreases in final glucose concentrations. If it is assumed that a plant operates at the highest possible solids concentration, the addition of recycling would either necessitate increasing the maximum operating solids concentration, which would require modifying process equipment, or leaving the solids concentration the same, which would lead to a decrease in starting cellulose content. However, the upper working limit of solids concentration in a plant may also be limited by enzyme inhibition and hydrolysate toxicity to the fermenting organism, so there may be room for adjustment in a specific process to incorporate solids recycle.

Interestingly, the increase in lignin in the insoluble fractions did not negatively impact the hydrolysis performance. With respect to washing of the solids, we did observe a tendency for improved glucose yields and thus better enzyme productivity with washing of the solids being recycled (Figure 2). These impacts of recycle could lead to significant necessary process modifications if enzyme recycling were to be implemented in an industrial process. Most importantly, in order to recycle some or all of the residual solids, tank size would have to increase, and different methods of mixing might have to be applied to the higher solids slurry. In addition, although substrate washing is currently not considered feasible for industrial lignocellulosic reactions, several issues may also have to be considered in relation to washing of the solids during the recycling, regardless of whether the washing includes specific variations such as recovery of the enzymes associated with the solids followed by concentration and new addition to the fresh substrate [19] or addition of surfactant as part of the solids washing and recycling [24]. In addition, using ammonia-pretreated corncob as cellulosic substrate it has recently been affirmed that cellulase adsorption kinetics to the substrate depends on the pH and that adsorption seems to be maximal around pH 4.8 [25]. Regardless of whether these latter results are considered or not, the cost of solid liquid separation to remove the insoluble solids would have to be added and recycling enzymes would thus lead to significant increases in invested capital costs of a plant. It remains to be seen if the decreased enzyme usage could offset the increased capital cost needed for the extra processing equipment and tank space.

Enzyme dosage could be decreased by 30% to achieve the same amount of cellulose hydrolysis in a steady state condition for the best conditions of the solids recycle experiments. This is a significant decrease in enzyme usage. It was also shown that solids recycle coupled with higher dosages of enzyme could improve yields beyond the baseline of no recycle level. It is worth noting though that the initial enzyme loadings for this study were set so that glucose yields would not approach theoretical maximum levels (below 85% total cellulose conversion), so that the effects of enzyme recycle could be observed in both the positive and negative direction from the control no recycle condition. Thus specific recycle performance results may vary based on what the desired level of cellulose hydrolysis is for a given process. As well, these varying results may impact process economics to the extent that solids recycle may or may not be economically beneficial. The enzyme recycle could be coupled with other methods for improving hydrolysis performance (enzyme dosage, reaction time) which might enhance recycle performance. The solids recycle method was shown to be an effective way to decrease enzyme usage, however its specific application in a continuous industrial process has yet to be proved, and may vary greatly depending on the process applied. Enzyme recycle can be effective in decreasing enzyme usage, however its overall benefit to a specific process must be tested at a larger scale.

## Conclusions

It was possible to effectively recycle a significant amount of the enzymes in Cellic CTec2 by recycling the insoluble solid fraction after enzymatic hydrolysis of pretreated corn stover. Enzyme usage was decreased 30% while maintaining glucose yields when a majority of the insoluble material was recycled. Similar trends in the recyclability of the enzyme were seen at two total solids concentrations, suggesting that this process could be replicated at even higher total solids operating conditions. As well, specific enzyme productivity increased for all recycle scenarios.

This represents a significant improvement in process performance and a significant reduction in the enzyme requirement for lignocellulose hydrolysis. However, by recycling the insoluble fraction, total solids concentrations, reaction masses, and the amount of lignin in the reaction increased significantly, and may therefore temper improved process performance and economics. This balance will be highly dependent on a specific industrial process and operating costs. Elevated levels of lignin residue were not found to negatively affect enzyme performance. Enzyme recycle remains a viable method for decreasing enzyme requirements and operating costs for renewable fuel production, and its industrial application should be further investigated.

## Materials and methods

### Feedstocks and chemicals

Dilute acid pretreated corn stover (PCS) used as the model substrate in this study, and was kindly provided by the National Renewable Energy Laboratory (NREL; Golden, Colorado). The corn stover was pretreated using a dilute sulfuric acid pretreatment method in a continuous pilot scale pretreatment reactor [26]. Two batches of PCS were used for the study, and their compositions and gravimetric data are given in Table 1. Batch 1 (PCS1) was dewatered in a hydraulic press (0-900 psi, 1–5 minutes) to remove a majority of the soluble sugars, and batch 2 (PCS2) was washed thoroughly, and milled to allow for accurate pipetting of the biomass in small scale experiments. All of the reported experiments used batch 1 material except when evaluating the effect of increased recycled lignin rich material (Figure 10). Feedstock composition was determined using NREL’s standard Laboratory Analytical Procedures (LAP) for the determination of the composition of biomass [27]. All chemicals used in the study were reagent grade and purchased from Sigma-Aldrich (St. Louis, MO).

**Table 1:** Compositional analysis of lignocellulosic substrates used in the study

**Substrate**	**Glucan(w/w%)**	**Xylan(w/w%)**	**Acid insoluble lignin(w/w%)**	**Acid soluble lignin(w/w%)**	**Ash(w/w%)**
NREL Pretreated corn stover (batch 1)	62.1	2.4	25.2	1.6	2.7
NREL washed pretreated corn stover (batch 2)	57.5	7.0	27.2	NA	6.5

### Cellulase enzyme

Enzymatic hydrolysis was carried out using Cellic CTec2, a commercial enzyme preparation kindly provided by Novozymes (Bagsværd, Denmark). Enzyme was loaded on a mass of enzyme product (EP) per gram of glucan (cellulose) in the substrate basis.

### Enzymatic hydrolysis procedure

Enzymatic hydrolysis was carried out except where noted with an initial total reaction mass of 20 g, and an initial biomass concentration of between 10-15% TS (w/w) on a dry weight basis. Samples were prepared by adding deionized water (Millipore® Milli-Q, USA), sodium citrate buffer (50 mM final citrate buffer concentration), and sodium hydroxide for pH adjustment (4 N) to the correct amount of biomass substrate, loaded on a dry weight basis. Initial pH was between 5.1 and 5.3 for all samples. Three stainless steel 4 mm ball bearings were added to each sample to facilitate mixing during the reaction. All reactions were carried out at 50°C (+/− 0.5°C) for 72 hours in a rotisserie style incubator (Combi-D24 hybridized incubator, from FINEPCR, Korea) rotating end over end at approximately 30 rpm. The reactions were carried out in batch mode for each recycle round.

### Recycle procedure

Enzyme recycle was carried out by recycling the insoluble solid residue present at the end of each hydrolysis period. For each recycle round the insoluble solids residue from the previous batch hydrolysis was mixed thoroughly with 20 g of fresh substrate slurry as the original initial % TS for the experiment. The recycle followed one of two similar methods, based on whether or not the solids were washed between recycles (Flow chart, Additional file 1). After hydrolysis for 72 hours, the samples were centrifuged at 4000 relative centrifugal force (RCF) for 20 minutes at 20°C to separate the solid and liquid fractions. The supernatant liquor was decanted and glucose concentration was measured. If the solids were to be washed according to the experimental design, 35 ml of deionized water was mixed thoroughly with the insoluble solids, centrifuged again and the wash liquid decanted. 20 g of fresh substrate slurry, prepared in the same manner as the initial hydrolysis was mixed with the desired amount of insoluble solids to be recycled. Additional Cellic CTec2 was added to the mixture and loaded based on the amount of cellulose added to the experiment from the fresh substrate slurry. Glucose concentration was measured in the new mixture at time zero to account for glucose carried over from the previous hydrolysis. The process was then repeated for each subsequent recycle round.

### Experiments

#### Resuspension of residual solids experiment

The standard enzymatic hydrolysis was carried out for 72 hours with initial 15% TS. The samples were then centrifuged and the liquid fraction decanted, and either citrate buffer, buffer with 34 mg EP/g fresh cellulose calculated based on the addition of 20 g fresh substrate, or 20 g fresh substrate at 15% TS with no additional enzyme was added to the samples. Samples were then incubated at 50°C for a further 72 hours after which glucose concentrations were measured. Glucose concentration was measured in the liquor fractions after the first initial 72 hours, after resuspension of the solids residue, and after the end of the reaction. Experiments were carried out in triplicate.

#### Factorial recycle experiment

A full factorial statistical design with two continuous variables and one categorical variable was designed to determine the effect on glucose production. The amount of insoluble solid material re-cycled into the subsequent hydrolysis was varied between 50% and 100% (w/w). The amount of makeup enzyme added was varied between 0 and 34 mg EP/g fresh cellulose added with the new slurry added in each recycle round. The categorical variable was the washing of the solids. Additional enzyme was applied only on the basis of the amount of new cellulose added, and not on the cellulose in the recycled insoluble solids. This experiment was carried out on batch 1 PCS with an initial 10% TS. A total of 4 recycle rounds were carried out, for a total reaction time of 360 hours. The initial enzyme loading at the start of the experiment for all conditions was 51.4 mg EP/g cellulose. The experiment was carried out in one batch with duplicate experiments, with the center point of the experimental design repeated 6 times. The data were statistically analyzed using least squares fit modeling and surface response fit to determine the significance and effects of each factor. Experiments were designed and analyzed using SAS JMP software (SAS Inc., Cary, North Carolina). Significance was defined as probability value (P values) below 0.05 for type I errors.

#### Response surface recycle experiment with higher solids concentrations

The initial substrate solids concentration was increased to 15% TS for the second round of statistical experiments. The experiment was designed as a response surface central composite design to determine the impact of fraction of insoluble solids recycled, varied from 70-96%, and additional enzyme loading, which varied between 23–46 mg EP/g fresh cellulose. 3 recycle rounds were carried out for this experiment, for a total reaction time of 288 hours. The initial enzyme loading for all conditions was 51.4 mg EP/g cellulose. The center point was repeated 6 times and the rest of the experimental conditions were carried out in duplicate experiments.

#### Lignin residue hydrolysis response

Lignin rich hydrolysis residue was produced by hydrolyzing batch 1 PCS with 51.4 mg EP/g cellulose for 144 hrs at 10% TS. This resulted in a residual insoluble solids material that consisted of 95% lignin and 5% residual cellulose. The material was thoroughly washed before addition. The lignin material contained residual enzyme activity, and it was applied both before and after a deactivation step (10 minutes at 100°C in a block heater (VWR, USA). A volume of 800 μl of batch 2 PCS at 6.25% TS in citrate buffer (50 mM) was added to 2.5 ml reaction tubes. The lignin residue was added in varying amounts to the substrate (0-60% of total solids w/w). Enzyme loading was 34 mg EP/g cellulose based on the cellulose in the substrate. The tubes were placed in a tabletop shaker (Thermo Mixer Comfort, Eppendorf, Hamburg) temperature controlled at 50°C and mixed at 1300 rpm. Glucose concentrations were measured after 72 hours. Experiments were carried out in duplicate.

### Analytical methods

#### BCA protein analysis

The protein concentrations in the enzyme preparation were measured using the bicinchoninic acid (BCA) analysis assay method which has been described previously (Smith et al., 1985) [28]. A BCA assay kit from Thermo Scientific was used for the analysis (Walthman, USA).

#### Sugars and dry matter analysis

Sugar concentrations were measured using High Performance Liquid Chromatography (HPLC). The HPLC used was a Waters HPLC 2695 with a Biorad-H 87+ column (Waters Corporation, Millford, USA). The eluent was 5 mM sulfuric acid with a flow rate of 0.5 ml/min. Percent solids (%TS) measurements were made using an 105 °C oven (Memmert, Germany) method according to standard procedures developed at NREL [27].

### Calculations

#### Glucose yield

Glucose yields, *Y*_glucose_, (equation 1, Table 2), were calculated by measuring the glucose concentration in the liquor fraction after each hydrolysis round, and estimating liquor volume after hydrolysis to determine the mass of glucose produced [29]. This was compared to the total cellulose content of the substrate (modified by a hydration factor of 1.11) while discounting any soluble glucose present at the start of the reaction. Glucose concentration was thus measured in the liquid fraction at the beginning of each recycle round (*C*_*gluc initial*_), and this concentration was subtracted from the final concentration of glucose (*C*_*gluc final*_) to avoid double counting (equation 1, Table 2).

**Table 2:** Equations used for calculating glucose yield, *Y*_**glucose **_(% g glucose/g cellulose) (equation 1) and enzyme productivity, *P*_**enztot **_(g glucose/mg enzyme protein) (equation 2)

	**Equation**
*Y*_glucose_ (equation 1)	$Y_{\text{glucose}}=\frac{C_{\mathrm{gluc}\;\mathrm{final}}-C_{\mathrm{gluc}\;\mathrm{initial}})\cdot V_{\mathrm{final}}}{M_{\mathrm{cellulose}\;\mathrm{tot}}\cdot 1.11}$
*P*_enztot_ (equation 2)	$P_{\mathrm{enztot}}=\frac{\sum_{0}^{N}\left(C_{\mathrm{gluc}\;\mathrm{final}}^{n}-C_{\mathrm{gluc}\;\mathrm{initial}}^{n}\right)\cdot V_{\mathrm{final}}^{n}}{\sum_{0}^{N}M_{\mathrm{enz}}^{n}}$

For *Y*_glucose_ equation 1: The glucose yields were calculated based on the total present cellulose in each hydrolysis round. *C*_*gluc final*_ is the final glucose concentration in the liquor fraction, *C*_*gluc initial*_ is the initial glucose concentration, *V*_*final*_ is the final volume of the liquor fraction, and *M*_*cellulose tot*_ is the total mass of cellulose present at the beginning of the hydrolysis. For the recycle rounds this included both cellulose from the fresh substrate and cellulose which was carried over in the insoluble solids. The amount of cellulose carried over from the previous recycle round was calculated by subtracting the glucose produced in the previous recycle round and assuming conservation of cellulose in the system. The glucose yields were calculated based on the total present cellulose in each hydrolysis round. It was assumed that all cellulose not converted to glucose remained evenly distributed with the insoluble fraction (for the 15% TS experiment, the amount of liquid removed was approximately 12 g (± 2 g) depending on recycle conditions. This amount was only a function of the efficiency of centrifugation).

For *P*_*enztot*_ equation 2: *M*_*enz*_^*n*^ is the mass of enzyme protein added for each recycle round *n*, and *N* is the total number of recycles. The other symbols are as explained for equation 1.

Total glucose yield was calculated based on total added cellulose during the course of the whole experiment and the mass of glucose produced over that same period. This reflected the glucose yield for the entire experiment, and controlled for varying levels of solids recycle and glucose removal during the recycles.

#### Enzyme productivity

The enzyme efficiency or productivity, *P*_*enztot*_, as it is referred to here, was calculated as a classic biocatalytic productivity term from the sum of all glucose produced during the course of the entire experiment divided by the total mass of enzyme protein product added during the experiment, with units of g glucose/ mg enzyme protein and calculated as shown in equation 2, Table 2. Mathematical modeling and assumptions

A mathematical model was developed to predict reaction parameters under different recycle scenarios for extended recycle rounds beyond the limit of the experiments. The model was a mass balancing of the recycle experiments, and relied on batch kinetics and stepwise computation of forecast results. Experimental results from the recycle experiments were extrapolated to predict the composition of the substrate, the total solids concentration, and total reaction mass. This was done using calculations based on a mass and component balance of the system. It was assumed that the final glucose yields and sugar concentrations remained constant from the final experimental hydrolysis, that the efficiency of the solid liquid separation step remained constant, and that the input mass and%TS of the fresh substrate was constant throughout additional recycle rounds. The recycle scenario was modeled as a series of batch reactions with solids recycle taking place at the end of an unspecified reaction time. Calculations for the computation of selected forecast data are given in the Additional file 1. Model sensitivity was not calculated, but the differences in the experimental data provided for significant differences in the model outcomes.

## Abbreviations

BCA: Bicinchoninic acid; PCS: Pretreated corn stover; %TS: Percent total solids; EP: Enzyme product; NREL: National Renewable Energy Laboratory; RCF: Relative centrifugal force.

## Competing interests

Drs. Johan Börjesson and Lars S. Pedersen are employed at Novozymes A/S, a company that sells enzymes for lignocellulose processing. The authors declare that they have no competing interests.

## Authors’ contributions

NW and JB conceived the study. NW designed experiments, carried out the laboratory work, and wrote the manuscript. JB provided supervision and research direction of the experimental work, as well as editing the manuscript. LSP provided project supervision, helped with statistical design and analysis, and edited the manuscript. AM provided academic supervision and edited the manuscript. All authors contributed intellectually via scientific discussions during the work and have read and approved the final manuscript.

## Supplementary Material

Additional file 1 Process flow diagram for the recycle procedure for insoluble solids recycle. Click here for file
