# Supplementary material for: Enzymatic lignocellulose hydrolysis: Improved cellulase productivity by insoluble solids recycling
Source: Biotechnol Biofuels. 2013 Jan 21;6:5. doi: 10.1186/1754-6834-6-5 (PMC3560254; doi:10.1186/1754-6834-6-5)
Supplement: Additional file 1 — Process flow diagram for the recycle procedure for insoluble solids recycle. [file 1754-6834-6-5-S1.docx]

# Supplementary Material

Enzymatic lignocellulose hydrolysis: Improved cellulase productivity by insoluble solids recycling

Noah Weiss, Johan Börjesson, Lars Saaby Pedersen, Anne S. Meyer

**Process flow diagram for the recycle procedure for insoluble solids recycle**. A indicates the reaction mixture without substrate; B is the enzyme reaction step; C separation; D mixing in of fresh substrate. The washing step was applied between steps C and D if it was part of the experimental design.


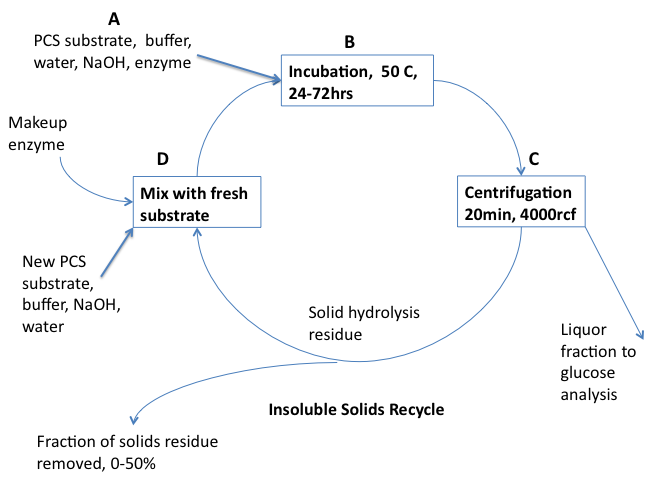


**Modeling of subsequent recycle steps**: The mathematical model applied was based on a mass balance of the system, where fresh pretreated material was added at the beginning of each recycle round, and material left the system as either soluble sugars in the liquid fraction, or as a fraction of an insoluble pellet (formed after centrifugation or some sort of solid liquid separation step) which was discarded at a certain fraction to maintain stability in the system. The weight fraction of the pellet which was recycled is referred to as %recycled (%R). Calculations were made in a stepwise fashion, using experimental data to populate the mass and component balances and then using the following equations to calculate values for the next recycle round. Values for the percent total solids (%TS) of a given recycle round (n) was calculated as:

$${\%TS}_{n}=\frac{{m^{n-1}}_{TS\_R\_pellet}\cdot\%R+{m^{n}}_{TS\_added}}{{m^{n-1}}_{pellet}\cdot\%R+{m^{n}}_{tot\_add}}$$

Where ${m^{n-1}}_{TS\_R\_pellet}$ is the mass of total solids in the pellet from the previous recycle (n-1), ${m^{n}}_{TS\_added}$ is the mass of total solids added at the beginning of each recycle round, ${m^{n-1}}_{pellet}$ is the mass of the total pellet remaining after solid liquid separation from the previous recycle round, and ${m^{n}}_{tot\_add}$ is the total mass of material added at the beginning of recycle round (n).

Lignin composition of the insoluble fraction was calculated via a similar method, such that the total fraction of lignin in the soluble solids was calculated to be:

$$\%lignin={\frac{m_{lignin}^{n-1}\cdot\%R+m_{lignin}^{n}}{{m^{n-1}}_{pellet}\cdot\%R+{m^{n}}_{tot\_add}}}$$

Where $m_{lignin}^{n-1}$ is the mass of lignin present in the previous recycle round (n-1), and $m_{lignin}^{n}$is the mass of lignin added to the recycle round (n) with the fresh material. It was assumed that lignin was inert during the hydrolysis, and therefore only left the system in the discarded solid fraction from the recycled material.
